# 2-DOF Small-Size Piezoelectric Locomotion Platform with the Unlimited Motion Range

**DOI:** 10.3390/mi12111396

**Published:** 2021-11-13

**Authors:** Andrius Čeponis, Dalius Mažeika, Vytautas Jūrėnas

**Affiliations:** 1 Department of Engineering Graphics, Faculty of Fundamental Sciences, Vilnius Gediminas Technical University, LT-10223 Vilnius, Lithuania; 2 Department of Information Systems, Faculty of Fundamental Sciences, Vilnius Gediminas Technical University, LT-10223 Vilnius, Lithuania; dalius.mazeika@vilniustech.lt; 3 Robotics and Piezomechanics Laboratory, Institute of Mechatronics, Kaunas University of Technology, LT-4424 Kaunas, Lithuania; vytautas.jurenas@ktu.lt

**Keywords:** 2-DOF platform, unlimited locomotion, piezoelectric actuator

## Abstract

This paper presents numerical and experimental investigations of a small size piezoelectric locomotion platform that provides unlimited planar motion. The platform consists of three piezoelectric bimorph plates attached to the equilateral triangle-shaped structure by an angle of 60 degrees. Alumina spheres are glued at the bottom of each plate and are used as a contacting element. The planar motion of the platform is generated via excitation of the first bending mode of the corresponding plate using a single harmonic signal while the remaining plates operate as passive supports. The direction of the platform motion controlled by switching electric signal between piezoelectric plates. A numerical investigation of the 2-DOF platform was performed, and it was found out that the operation frequency of the bimorph plates is 23.67 kHz, while harmonic response analysis showed that the maximum displacement amplitude of the contact point reached 563.6 µm in the vertical direction while an excitation signal of 210 V_p-p_ is applied. Prototype of the 2-DOF piezoelectric platform was made, and an experimental study was performed. The maximum linear velocity of 44.45 mm/s was obtained when preload force and voltage of 0.546 N and 210 V_p-p_ were applied, respectively.

## 1. Introduction

There is a high demand for planar positioning and locomotion platforms that can provide micro or nanoscale resolution, fast response, and high dynamic characteristics [1,2]. Such devices are used for laser systems and high-resolution imaging systems, micromachinery, biochemical applications, etc. Several types of locomotion platforms have been developed up till now that operate based on electromagnetic, electrostatic, and piezoelectric principles [3,4,5,6]. The majority of the platforms are driven by electromagnetic and electrostatic actuation principles. However, these types of platforms have a relatively complicated structure, limited scaling options and are sensitive to the external electric and magnetic fields. In addition, platforms based on electrostatic and electromagnetic actuators have limited resolution of motion as well as operation range [7,8,9]. Piezoelectric locomotion platforms can overcome these disadvantages and fulfill requirements of nanoscale resolution, scalable design, and fast response time. In addition, these platforms are magnetic and static field-free, have self-locking ability, and can provide several degrees of freedom using a single actuator [10,11,12,13,14,15]. Moreover, piezoelectric devices do not need a gearing mechanism, so backlashes do not occur in the system. Usually, piezoelectric positioning devices have a simple structure, straightforward operation principle as well as relatively uncomplicated control system. Therefore, these advantages forced the investigation of piezoelectric locomotion platforms and their application in different positioning systems [16,17].

Hernando-Garcia and et al. reported on piezoelectric bi-directional locomotion robot [18]. The robot was based on millimeter-sized rectangular glass plate with two piezoelectric patches, which were bonded at the ends of glass plate. The operation of the robot was based on traveling wave, which was generated at a frequency between resonances of two adjacent bending modes. On the basis of numerical and experimental investigation results, the authors claimed that the proposed robot was able to provide up to 100 mm/s speed while the excitation voltage was set to 65 V_p-p_ and 168 kHz. However, the proposed robot has disadvantages, such as: bi-directional planar locomotion motion can be generated only in one axis of motion, and in order to change the direction of motion by 90°, the robot should be manipulated manually. Moreover, the operation frequency of the robot is high and from electronics point of view, control and drive are complex.

Lobontiu et al. investigated piezoelectric planar locomotion robot based on single piezoelectric unimorph actuator. At both ends of the actuator are placed supports of custom design [19]. Piezoelectric inchworm operation principle is adopted for the planar locomotion of robot. Inchworm operation principle is obtained via different lengths of the supports. During vibrations of piezoelectric actuator, the supports generates different motion trajectories and as a result, planar motion of robot occurs. The robot has simple design, excitation schematics and good dynamic characteristics. However, the proposed design has disadvantages such as: motion direction cannot be changed during operation of the robot, it should be changed manually as well as complex designs of supports can effect motion resolution and reliability of whole system.

Becker et al. reported on a piezoelectric beetle-robot for planar locomotion [20]. The proposed robot is composed from triangle plate with legs, which are located at each corner of it. On top of the plate is placed a circular unimorph piezoelectric actuator, which acts as an active part of the robot. The robot was operated by using different vibration forms of excited triangular plate. The direction of motion was controlled via excitation frequency, i.e., by changing the vibration mode of the triangular plate. However, usage of different vibration modes to control motion direction makes robot control complex, as well as the speeds of motion in different directions will not be the same due to the discrepancies in vibration frequencies.

Fan et al. investigated a walking robot that was built with eight piezoelectric bimorph actuators [21]. The robot was composed by clamping the actuators to the robot frame under different angles of inclination. Different angles of inclination ensures possibility to obtain forward and backward motions of robot. Impact operation principle was adopted to drive the robot. Four actuators were set to create forward motion while left actuators were dedicated to generate backward motion. Considering to the results of numerical and experimental investigations authors concluded that the robot is able to generate planar motion up to 33.8 mm s^−1^ while 50 g load is applied. Despite to good dynamic characteristics and possibility to change motion direction by switching control signal between actuators, the robot has disadvantages such as bulky design, non–resonant operation principle and complex control of motion direction.

This paper introduces numerical and experimental investigation of a novel piezoelectric 2-DOF locomotion platform with unlimited motion range in the plane and high dynamic characteristics. The platform has a simple design and is based on three bimorph piezoelectric plates. A single harmonic signal is needed to actuate the planar motion of the platform. Control of motion direction is implemented by a simple digital switch box. The rest of the paper is organized as follows. Section 2 describes the structure, dimensions, and operation principle of the platform. In addition, excitation schematics of the electrodes are described. Section 3 presents the results of numerical modeling. Section 4 provides the results of the experimental study. Finally, Section 5 concludes this work.

## 2. The Design and Operation Principle of the Platform

**Figure 1:**
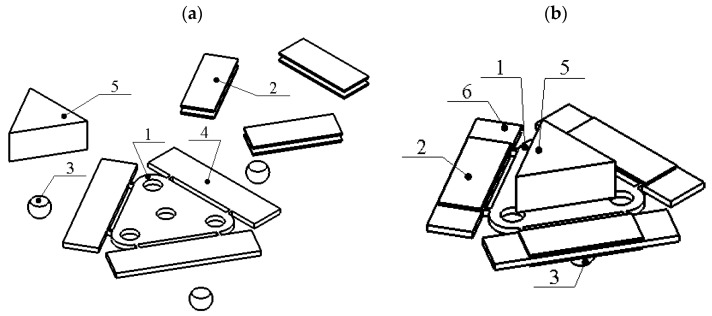
Design of the piezoelectric platform; (**a**)—exploited view; (**b**)—assembled view; 1—triangle shaped platform; 2—piezo ceramic plate; 3—spherical contact; 4—rectangular plate; 5—payload; 6—piezoelectric bimorph plate.

The proposed piezoelectric locomotion platform can move in plane. The displacement range of the platform is limited only by the size of the plane and the length of the wires used to connect piezoelectric actuators to power generator. The plane used for platform locomotion must be made of a hard material such as glass or alumina. The platform consists of three piezoelectric bimorph plates attached by an angle of 60 degrees to the edges of an equilateral triangle-shaped plate (Figure 1). The thin junction beams are used to compose piezoelectric bimorphs and triangle plate into a single platform. In order to reduce structural damping of bimorph plates, the beams are placed at the nodes of the first out of plane bending mode. Alumina spheres are glued at the bottom of each bimorph plate and are used as contacting elements between the platform and plane. The exploded and assembled views of the platform are shown in Figure 1. It can be seen that the triangle plate has four holes that are used to fix payload directly on the top surface of the platform. The payload can be placed on the top surface without fixing it as well (Figure 1b).

**Figure 2:**
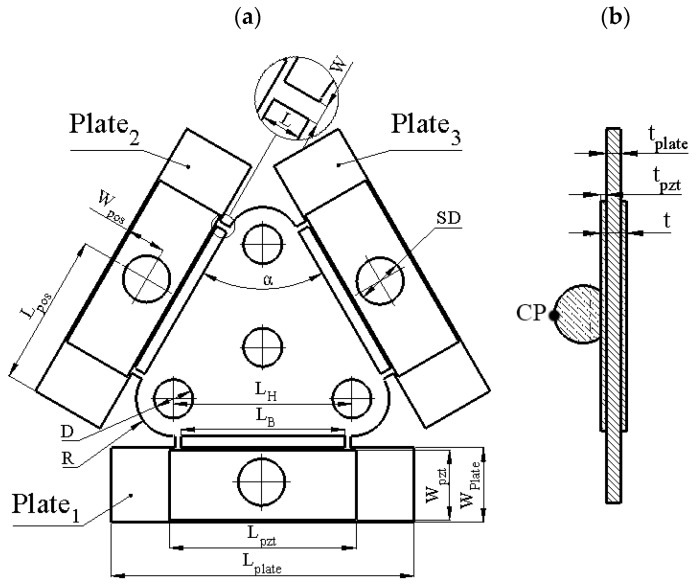
Sketch of the platform; (**a**)—bottom view; (**b**)—section view of the bimorph plate with the spherical contact; CP—contact point.

Dimensions of the piezoelectric platform are listed in Table 1, while Figure 2 explains notations used to describe the dimensions of the platform. It must be noted that the dimensions of the bimorphs and the position of the spherical contacts were obtained by solving optimization problems that are described in Section 3.

The operation principle of the platform is based on the first out of plane bending mode B_02_ of the rectangular-shaped bimorph plate. The modal shape of the bimorph plate is asymmetrical because of fixing of the plate to the platform, i.e., only one edge of the bimorph plate is fixed. Therefore, vibrations of the spherical contact are generated in two-dimensional space. Such vibrations induce curvilinear motion of the spherical contact in the plane that is perpendicular to the contacting plane and make an impact to the contacting surface. The platform generates motion via vibro-impact principle.

The design and operation principle of the piezoelectric platform provides possibility to achieve controllable planar motion of payload. The excitation of the platform is performed by applying a single harmonic signal to the particular piezoelectric bimorph plate. Such excitation inducts linear motion of the platform in direction aligned to the transverse symmetry axis of the active bimorph plate.

**Figure 3:**
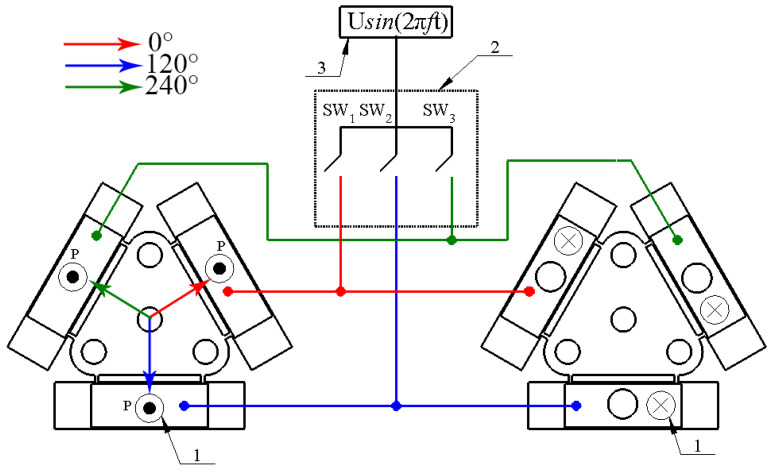
Excitation schematics of the platform; 1—direction of piezo ceramic polarization; 2—digitally controlled switch box; 3—signal generator.

The direction of the platform motion can be controlled by switching excitation signal between different bimorph plates using a digitally controlled switch box. Reverse motion into a particular direction can be obtained by applying the same electric signal to the electrodes of the other two bimorph plates. Excitation schematics and switch box control signals are given in Figure 3 and Table 2.

The trajectory planning algorithms and robust control can be applied to obtain the required planar motion direction and it accuracy [22,23,24,25,26,27]. Voltage amplitude and electric signal timing can be used as input parameters of the algorithms. In addition, the platform can be driven by applying burst-type electric signal. Such excitation regime allows to achieve motion with high resolution.

## 3. Numerical Investigation of the Platform

The numerical investigations of the platform were performed to obtain optimal geometrical parameters, indicate the electrical and mechanical characteristics of the proposed design. The finite element model (FEM) was built using Comsol Multiphysics 5.4 (COMSOL, Inc., Burlington, VT, USA) software. Firstly, material properties were defined in the model. Stainless steel was used for the passive layer of the platform, PIC155 material properties were used for piezo ceramic plates, and finally, alumina oxide ceramic properties were used for spherical contacts. Values of material properties are given in Table 3. The polarization direction of the piezoelectric plates was aligned to the thickness of plates while the passive layer of the platform was grounded. The model was analyzed without mechanical constraints and loads, i.e., the contacts as well as the whole platform were set to free condition. Therefore, effect of friction, mechanical loads, etc., were neglected in the model.

Firstly, optimization of the bimorph plate dimensions was made with the objective to maximize vibration amplitudes of the antinode while the contact sphere was fixed at the center of the plate, i.e., $L_{pos}=\frac{L_{Plate}}{2}$ and $W_{pos}=\frac{W_{Plate}}{2}$. We have fixed the size of the triangle-shaped platform (Figure 1) and defined the width of the plate *W_Plate_* and length of the plate *L_Plate_* as design variables (Figure 2a). The total displacement amplitude of the antinode in Y and Z directions was set as the objective of the optimization problem. The analyzed platform has a symmetrical structure, so only one bimorph plate was analyzed. The description of the optimization problem is given below:

(1) $$\underset{L_{Plate,}W_{Plate}}{\max}\left(\sqrt{u_{y}^{2}\left(L_{plate},W_{Plate}\right)+u_{z}^{2}\left(L_{plate},W_{Plate}\right)}\right);$$

subjected to

(2) $$L_{Plate}^{min}\leq L_{Plate}\leq L_{Plate}^{max};$$

(3) $$W_{Plate}^{min}\leq W_{Plate}\leq W_{Plate}^{max};$$

(4) $$f_{min}\leq f\leq f_{max}.$$

Here, *L_Plate_* is optimal length of bimorph plate; *W_Plate_* is optimal width of bimorph plate; *u_y_* is displacement component in *Y* direction, *u_z_* is displacement component in *Z* direction; $L_{Plate}^{min}$ and $L_{Plate}^{max}$ are the lower and upper limits of bimorph plate length; $W_{Plate}^{min}$ and $W_{Plate}^{max}$ are the lower and upper limits of bimorph plate width; *f* is a resonant frequency of the bimorph plate; *f_min_* and *f_max_* are the lower and upper limits of the analyzed frequency range.

**Figure 4:**
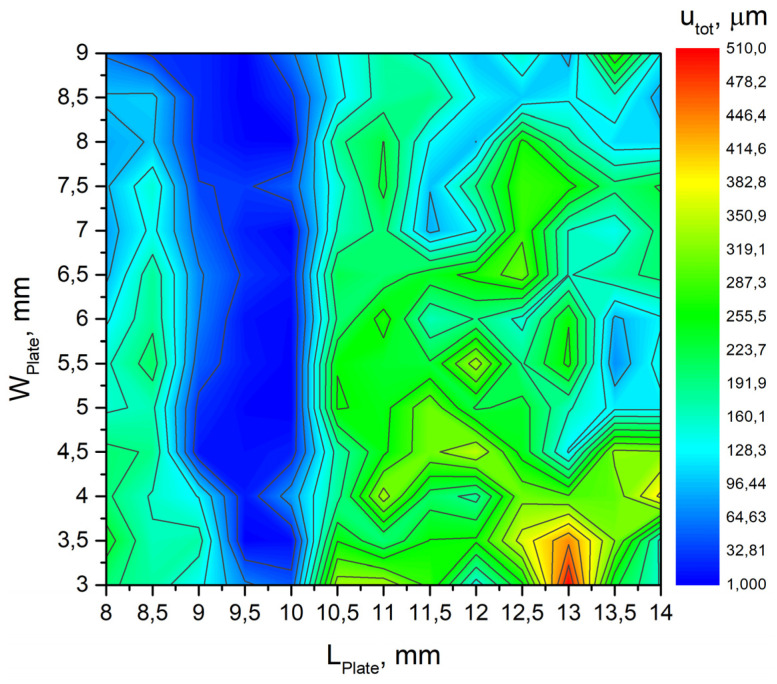
The plot of the objective function.

The lower and upper limits of bimorph plate length $L_{Plate}^{min}$, $L_{Plate}^{max}$ were set to 8 mm and 14 mm, respectively, while limits of the plate width $W_{Plate}^{min}$ and $W_{Plate}^{max}$ were set to 3 mm and 9 mm. An iteration size of 0.5 mm was used for both variables. The frequency range from *f_min_* = 20 kHz to *f_max_* = 52 kHz, and the step size of 5 Hz was determined. Frequency-response analysis of the platform was performed by applying a voltage of 210 V_p-p_ for the actuator excitation. The results of calculations are given in Figure 4, where the color legend represents the values of the objective function.

Analysis of the results revealed that the objective function has the maximum value of 508.12 µm when dimensions of the plate are *L_Plate_* = 13 mm and *W_Plate_* = 3 mm. The resonant frequency of the platform is 23.54 kHz. These dimensions of the plates will be used for further numerical and experimental investigations of the platform. Additionally, it can be found that optimum of the function is at lower limit of *W_plate_* variable. The calculations were not performed with lower values of this variable due to limits of practical implementation, i.e., limits of accessible manufacturing and piezo ceramic cutting processes. In addition, limits of the manual assembly process had influence to lower limit definition.

It was already shown that bimorph plates have asymmetrical clamping, so amplitudes of B_02_ bending vibrations are distributed in different directions compared to the case with symmetrical plate clamping. In order to determine the optimal position of the contact sphere along the transverse symmetry axis of the bimorph plate, the next optimization problem was formulated with the objective to maximize total displacement amplitude of the contact sphere in *Y* and *Z* directions:

(5) $$\underset{W_{pos}}{max}\;\left(\sqrt{u_{y}^{2}\left(W_{pos}\right)+u_{z}^{2}\left(W_{pos}\right)}\right)$$

subject to:

(6) $$W_{pos}^{min}\leq W_{pos}\leq W_{pos}^{max}$$

**Figure 5:**
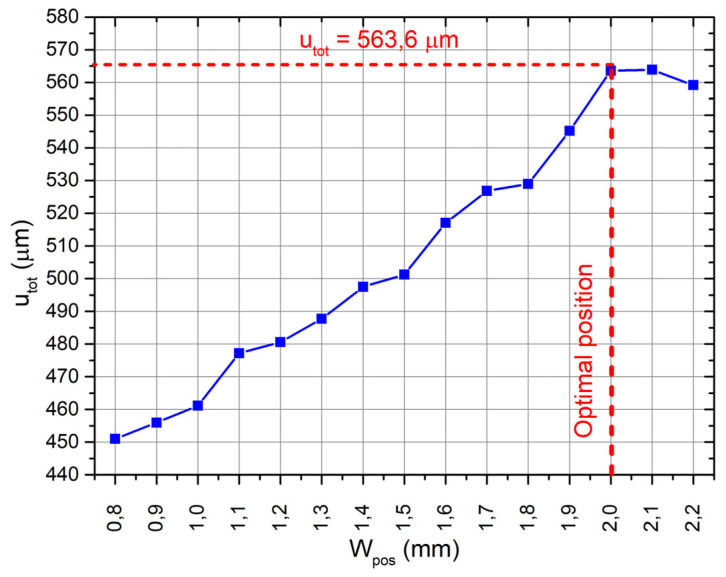
The plot of objective function of sphere position.

Here, *u_y_* and *u_z_* are displacement amplitude in the directions of the *Y* and *Z* axis, respectively; *W_pos_* is the distance of the contact sphere from the outer edge of the plate; $W_{pos}^{min}$ and $W_{pos}^{max}$ are the lower and upper limits of spherical contact position, respectively. The following values for lower and upper limits were used: $W_{pos}^{min}$ = 0.8 mm, $W_{pos}^{max}$ = 2.2 mm. Iteration size was set to 0.1 mm. The initial position of the sphere contacts were set to *W_pos_* = 0.8 mm. The FEM model with already optimized dimensions of the bimorph plates was used. Frequency-response analysis of the platform was performed by applying a voltage of 210 V_p-p_ as in the previous case. The dependence of the objective function from contact sphere position on the plate is shown in Figure 5.

As can be found in Figure 5, the optimal position *W_pos_* of the spherical contact along the transverse axis of the plate is 2 mm from the outer edge of the bimorph plate. The total displacement amplitude of the contact sphere is 563.6 µm at this position. It can be noticed that optimization ensured increment of amplitude by 10.91%compared to the value obtained at the first stage and showed that contact sphere should be located closer to the fixed edge of the bimorph plate. This increment is the result of more curved deformation of the plate closer to the fixed edge, which occurred due to unsymmetrical clamping. A summary of the results obtained from the optimization problems is given in Table 4.

**Figure 6:**
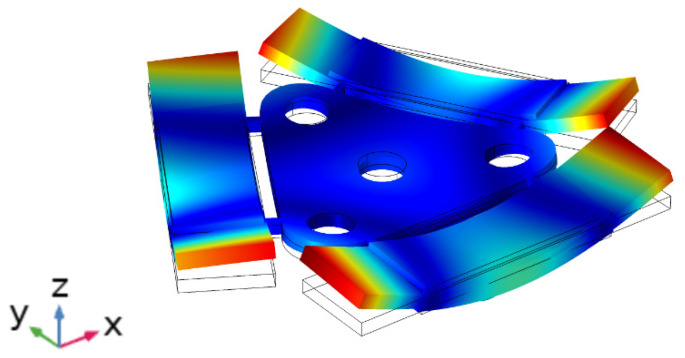
Modal shape of platform obtained at 23.54 kHz.

The next step of the numerical study of the piezoelectric platform was to perform modal frequency analysis. Mechanical boundary conditions were not applied. The modal shape of the platform is shown in Figure 6. It can be seen that bimorph plates have modal shapes similar to the first out-of-plane bending mode. Moreover, the bimorph plates vibrate not only in out of plane direction Z, but also in a horizontal direction because of asymmetrical clamping. Such vibrations of the bimorph plate allow us to achieve bias stroke of the contacting sphere and generate normal and tangential forces to the contact surface.

**Figure 7:**
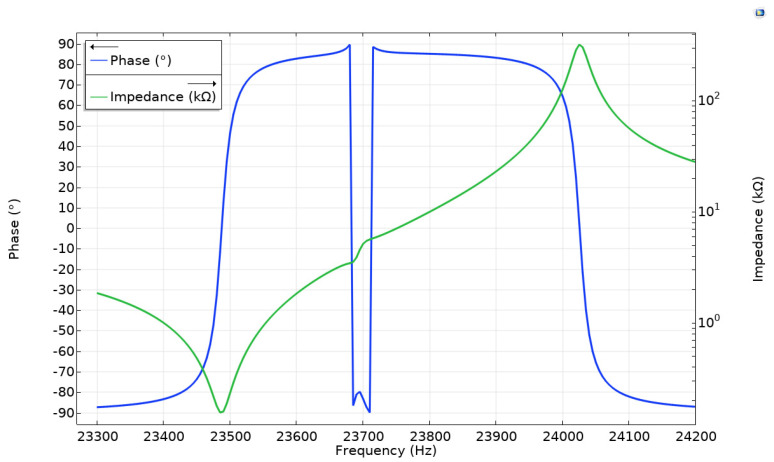
Impedance and phase frequency characteristics of the platform.

The further step of the numerical study was to analyze impedance and phase frequency characteristics of the platform in the frequency range from 23.3 kHz to 24.2 kHz with the step of 5 Hz (Figure 7). The study was performed when electrodes of Plate_2_ and Plate_3_ were set to open circuit condition while the harmonic electric signal with 210 V_p-p_ amplitude was applied to both electrodes, which were connected in parallel, of Plate_1_. The resonant frequency of 23.48 kHz was obtained and it confirms results obtained during the modal analysis. The minor mismatch between resonant frequencies occurs due to different electrical boundary conditions used in the model, i.e., modal analysis was performed when open circuit condition was applied to all bimorph plates. Additionally, it can be seen that the lowest impedance value of 161 Ω was obtained at the resonant frequency. Moreover, the calculated mechanical quality factor of the platform is *Q_m_* = 1398.1, while the effective coupling coefficient *k_eff_* = 0.0013.

**Figure 8:**
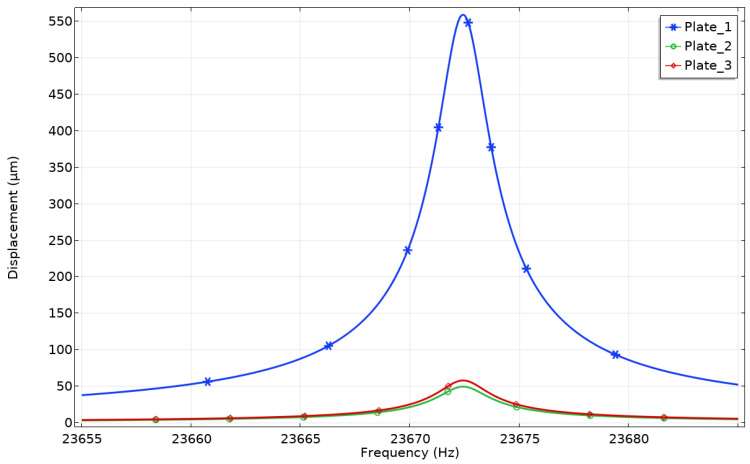
Displacement-frequency characteristics of the platform while Plate_1_ is excited.

A numerical analysis of the harmonic vibrations of the platform was made as the next step of the study. Vibrations of the three contact points (CP) were analyzed when only one bimorph plate was effected by electrical signal. Such an excitation regime is the most common for platform driving. It was important to study the coupling effect between vibrations of the excited bimorph plate and vibrations of the two remaining passive plates. Vibrations of unexcited plates can cause deviations from the desired motion direction of the platform. The frequency range from 23.655 kHz to 23.685 kHz was analyzed with a step resolution of 0.5 Hz. Figure 8 shows the total displacement amplitudes of all three contact points in the frequency domain when an excitation voltage of 210 V_p-p_ was applied to the single bimorph plate—Plate_1_.

**Figure 9:**
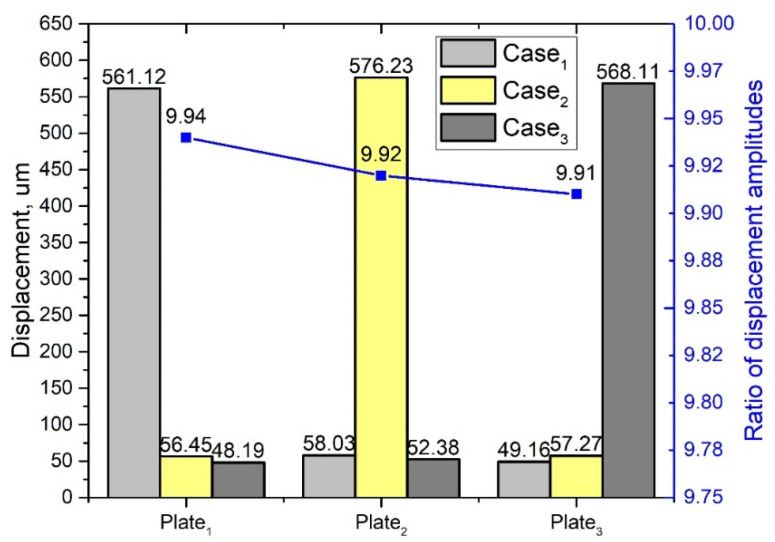
Summary of displacement amplitudes while different excitation cases are used.

It can be seen that the contact point (CP) located on the exciting plate has a displacement amplitude of 561.12 µm while contacts points (CP) located on Plate_2_ and Plate_3_ plates have 56.45 µm and 48.19 µm displacement amplitudes, respectively. Differences between displacement amplitudes of passive plates is caused by anisotropic properties of piezo ceramics, which leads to discrepancies of plate stiffness. The coupling ratio between displacement amplitudes is up to 10 times. The comparison of the vibration amplitudes and coupling ratio between amplitudes values is provided in Figure 9. It represents the three excitation cases used to generate forward motion of the platform (Table 2). It can be seen that coupling ratios between displacement amplitudes slightly fluctuate for different excitation cases, but these differences mainly come due to computing errors. Simulation results shows that the vibration amplitudes of the contact points (CP) located on the passive bimorph plates are significantly smaller compare to the amplitudes of vibrations of the sphere located on the active plate. Based on simulation results, it can be concluded that vibrations of the passive contact sphere moves the platform in the opposite direction compared to the active, but the force they generate is significantly smaller than that of the contact point (CP) located on the active plate.

**Figure 10:**
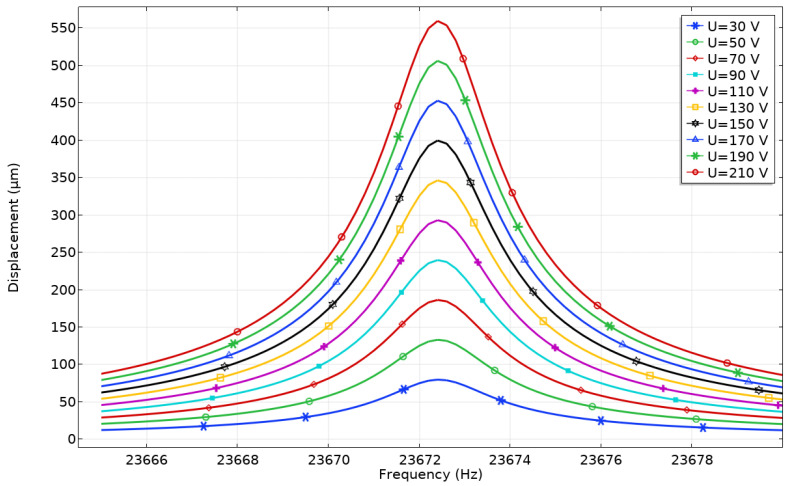
Displacement—frequency characteristics of the platform while different excitation voltages are applied to Plate_1_.

The dependence of displacement and velocity amplitudes of the contact point (CP) from excitation voltage were investigated in the frequency domain. The study conducted only when Plate_1_ was excited (Table 2, Case_1_, forward). The analyzed voltage range was set as follows: the lower limit of excitation voltage was set to 30 V_p-p_, the upper limit of excitation voltage was set to 210 V_p-p_ while an increment of 20 V_p-p_ was used. The displacement amplitude-frequency characteristic shows peaks of displacement amplitude that were obtained at the frequency of 23.673 kHz (Figure 10). The displacement amplitude reached 561.12 µm and the ratio between contact point (CP) displacement amplitude and the applied voltage is 2.67 µm/V_p-p_. On the other hand, minimum displacement amplitude was obtained at the same frequency while excitation voltage was 30 V_p-p_. The displacement amplitude reached 81.25 µm and the ratio of 2.71 µm/V_p-p_. It can be seen that almost linear dependence between displacement amplitude and excitation voltages was indicated. Therefore, control of the platform motion can be implemented using a simple electronic control system.

**Figure 11:**
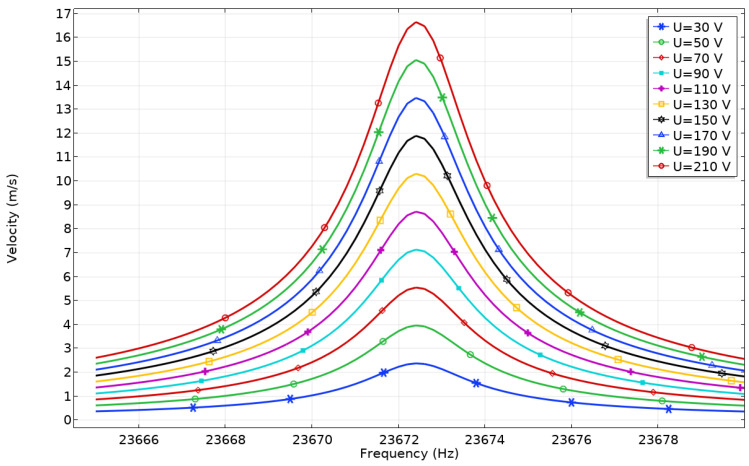
Velocity—frequency characteristics of the platform while different excitation voltages are applied to Plate_1_.

Further, contact point (CP) velocities at the different excitation voltages were analyzed (Figure 11).

Figure 11 shows that peaks of contact point (CP) velocity are obtained at the same resonant frequency as in case before. The highest velocity amplitude of the contact point (CP) of 16.65 m/s was obtained when the excitation voltage was 210 V_p-p._ The ratio between contact point (CP) velocity and the applied voltage is 79.2 mm/s/V_p-p_. The lowest velocity of contact point (CP) was obtained when excitation voltage was set to 30 V_p-p,_ and it reached 2.43 m/s or 81.12 mm/s/V_p-p_. It can be seen that an almost linear dependence between velocity and voltage is obtained.

**Figure 12:**
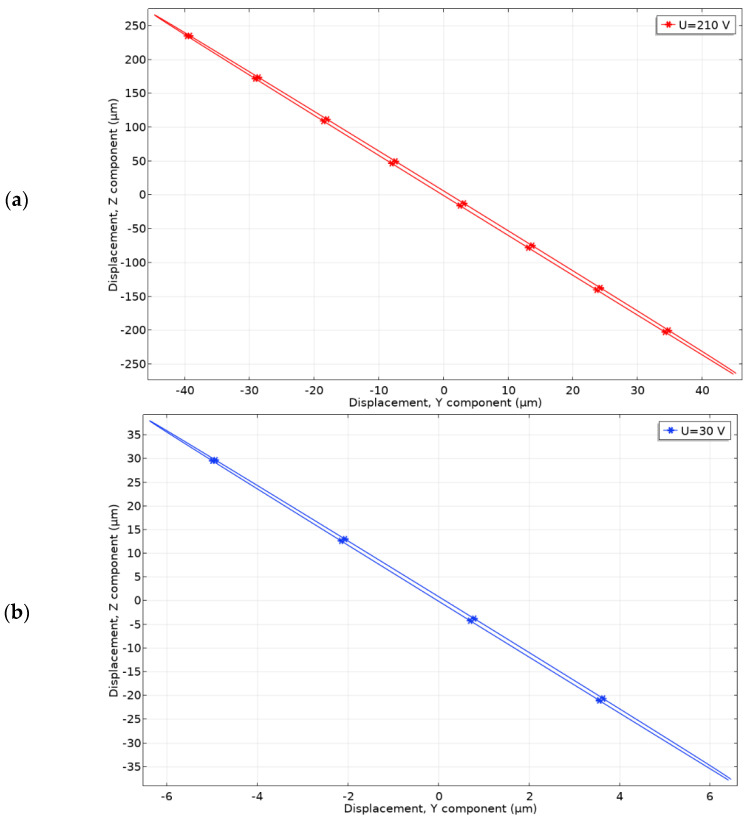
Motion trajectories of contact point located on Plate_1_; (**a**)—motion trajectory at 210 V_p-p_ excitation amplitude; (**b**)—motion trajectory at 30 V_p-p_ excitation amplitude.

Further, contact point (CP) motion trajectory was studied when excitation voltages of 210 V_p-p_ and 30 V_p-p_ were applied (Figure 12). Vibrations in the time domain were studied using the same numerical model at the frequency of 23.67 kHz. During analysis of results, it was noted that contact point (CP) moves in YZ plane and has an almost linear trajectory of motion.

The trajectory projection in X axis is 94.68 µm and 12.71 µm at the voltage of 210 V_p-p_ and 30 V_p-p,_ respectively. The same projections to *Y* axis are 541.12 µm and 76.84 µm. On basis of the results, it can be concluded that vibrations in Z direction are 5.71 times larger compared to the vibrations in *Y* direction, so friction force generated in the contact zone will be significant to drive the platform.

## 4. Experimental Investigation of the Platform

**Figure 13:**
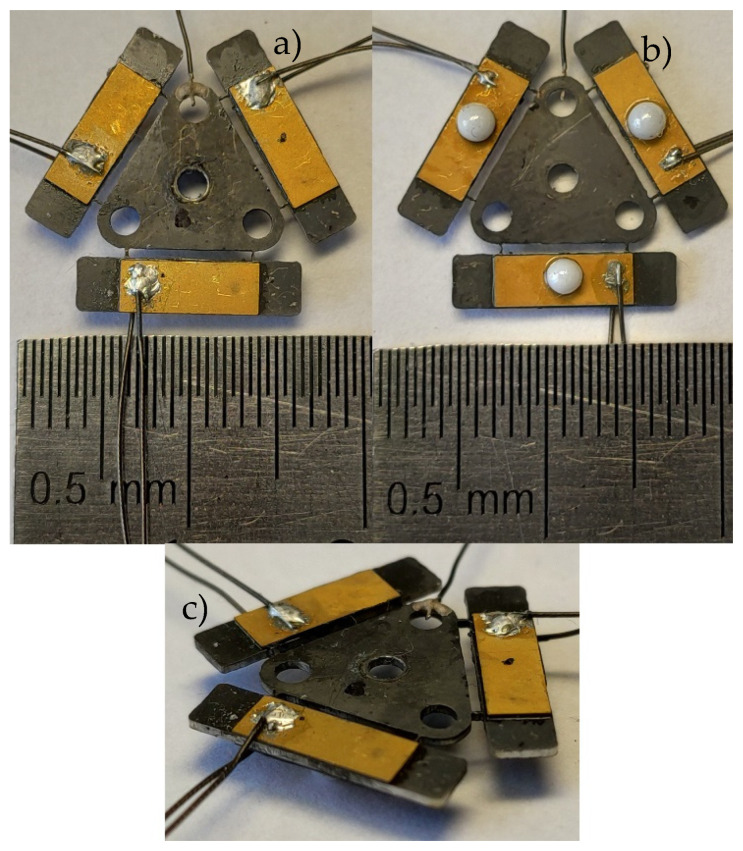
Prototype of the platform; (**a**)—top view, (**b**)—bottom view, (**c**)—side view.

The prototype of piezoelectric platform was fabricated for experimental study with strict respect to geometrical and physical parameters that were used and indicated during numerical investigations. The top, bottom, and side views of the prototype are shown in Figure 13.

**Figure 14:**
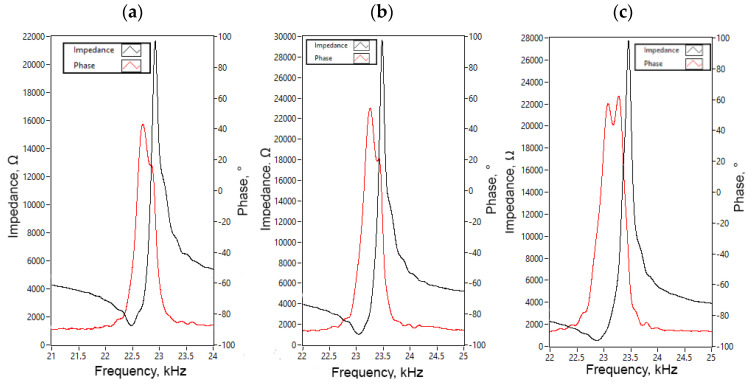
Impedance and phase frequency characteristics of the platform; (**a**)—characteristics of Plate_1_; (**b**)—characteristics of Plate_2_; (**c**)—characteristics of Plate_3_.

Impedance and phase frequency characteristics of the bimorph plates were measured using SinPhase 16777k (SinPhase, Moedling, Austria) impedance analyzer. Measurement of each bimorph plate was measured separately, while piezo ceramics plates of measured bimorph were connected in parallel. The remaining two bimorphs were set to open circuit condition. The results of measurements are given in Figure 14.

Experimentally, indicated resonant frequencies showed that minor differences, up to 570 Hz, between resonant frequencies of different bimorph plates occurs. The resonant frequencies of Plate_1_, Plate_2,_ and Plate_3_ are 22.47 kHz, 23.04 kHz and 22.86 kHz, respectively. Moreover, the difference between calculated and measured resonant frequencies is up to 5.3%. The impedance values of the plates at resonant frequencies are 993.62 Ω, 912.7 Ω, and 948.16 Ω, respectively, and fluctuation between values does not exceed 9%. Mismatches between results of numerical and experimental investigations mainly caused by manufacturing and assembly errors, neglected glue layer, and minor mismatches in material characteristics.

**Figure 15:**
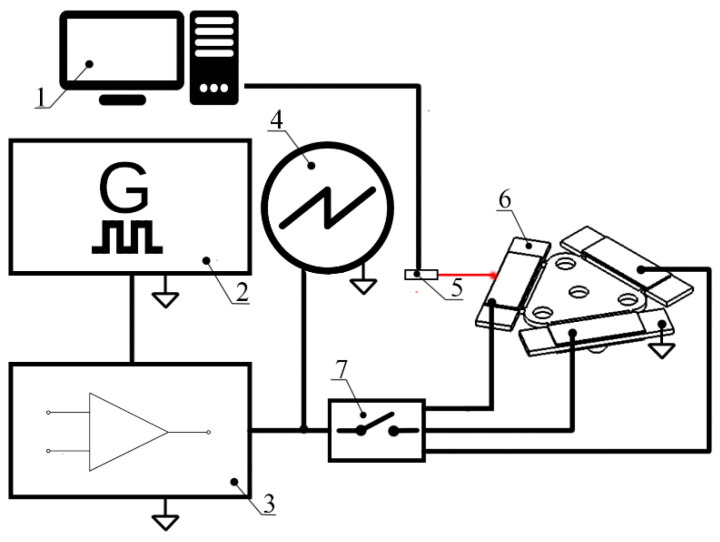
Scheme of the experimental setup: 1—a computer with data acquisition and switch control software; 2—function generator; 3—power amplifier; 4—oscilloscope; 5—displacement sensor; 6—a prototype of the actuator; 7—digitally controlled switch box.

Further, measurements of linear velocity were performed in order to investigate the dynamic characteristics of the platform when different excitation voltages and preloads are applied. The experimental setup was build, as is shown in Figure 15.

**Figure 16:**
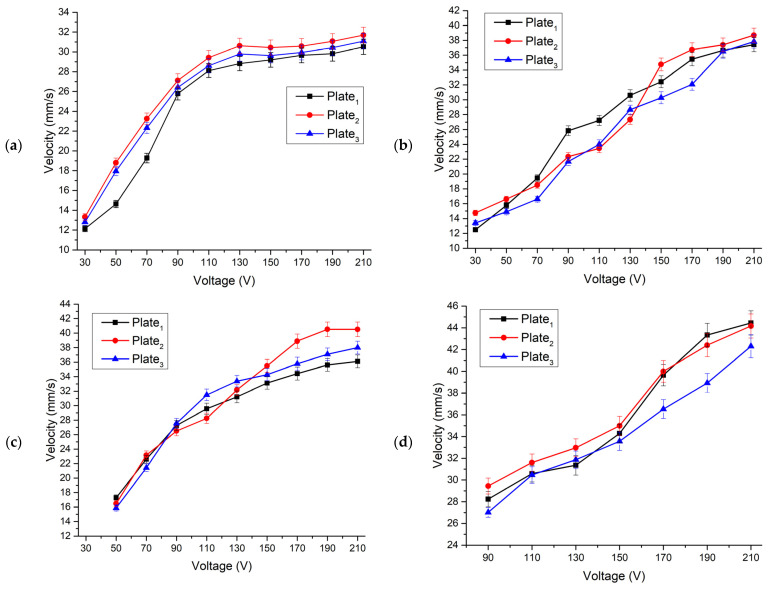
Velocity characteristics of the platform at different excitation voltages and load conditions; (**a**)—velocity characteristics while the load is 6.96 g; (**b**)—velocity characteristics while load is 12.05 g; (**c**)—velocity characteristics while the load is 32.17 g; (**d**)—velocity characteristics while load is 55.68 g.

The experimental setup consisted of a computer, a function generator WW5064 (Tabor Electronics, Israel), a power amplifier PX-200 (Piezo Drive, Shortland, Australia), oscilloscope DL2000 (Yokogawa, Tokyo, Japan), a displacement sensor ILD 2300 (Micro-Epsilon, Ortenburg, Germany) and a self-made switch box. The linear velocity of the platform was measured by varying the excitation voltage from 30 V_p-p_ to 210 V_p-p_ when four different loads of 6.96 g, 12.05 g, 32.17 g, and 55.68 g were applied (Figure 16). Velocity was measured when each bimorph plate was excited individually. It must be noted that startup voltage depends on the preload and increases when payload value is increasing (Figure 16).

The highest velocities were obtained when the voltage of 210 V_p-p_ was applied. The highest velocity generated by Plate_1_ reached a value of 31.07 mm/s or 0.147 mm/s/V_p-p_ when the load of 6.96 g was applied. The highest velocities of the Plate_2_ and Plate_3_ are 4–5% larger at the same load condition. When the load was increased up to 12.05 g, the velocity of the platform driven by Plate_1_ reached 37.82 mm/s or 0.18 mm/s/V_p-p_. Velocity values obtained during excitation of Plate_2_ and Plate_3_ at the same load conditions, varies in the range of 8–12%.

**Figure 17:**
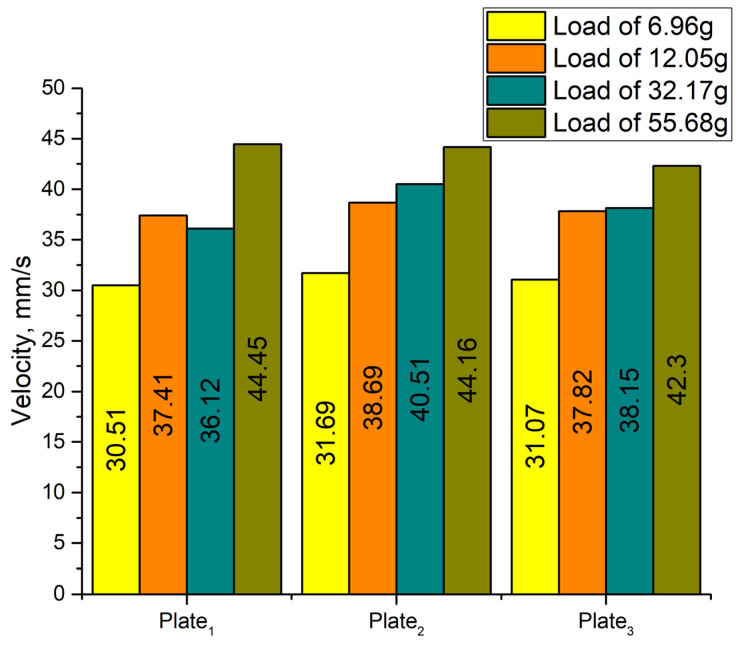
Summary of maximum velocity values at different load conditions.

Finally, during the analysis of velocity characteristics while loads were equal to 32.17 g and 55.68 g, it was found that the highest values were obtained at 210 V_p-p_ excitation voltage. The velocities reached 38.15 mm/s and 44.45 mm/s or 0.182 mm/s/V_p-p_ and 0.212 mm/s/V_p-p_, respectively. The difference between velocities does not exceed 10–15%. It can be concluded that the highest velocity was obtained at the highest load that ensured the highest friction force between the surface and contact point (CP) of the platform. However, the startup voltage was 90 V_p-p_ in this case. It makes the platform application less flexible compared to cases with lower loads. The summary of the highest velocities is shown in Figure 17.

**Figure 18:**
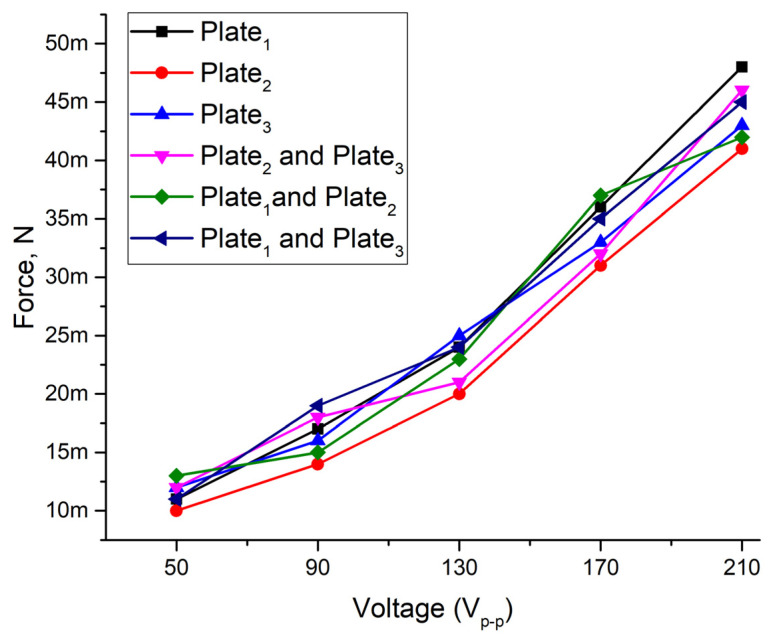
Output force characteristics at different excitation voltages while 32.17 g load was applied to the platform.

Thrust force measurements of the platform were performed when the load of 32.17 g was applied, and excitation voltage was changed in the range from 50 V_p-p_ to 210 V_p-p_. The measurements were performed for each bimorph plate separately and for all possible combinations of two plates. The results are given in Figure 18. It can be seen that thrust force increases when excitation voltage increasing. The lowest average thrust force of 11.5 mN was obtained when the voltage of 50 V_p-p,_ was applied. The deviation is 5.5 µN. The ratio of average output force to excitation voltage is 200 µN/V_p-p_.

The highest thrust force values were obtained at 210 V_p-p,_ while the average force value is 44.16 mN. The ratio of the average value to excitation voltage is 210 µN/V_p-p_. Results also shows that thrust force almost linearly depends on the applied voltage amplitude. Moreover, the difference between output forces generated by different plates and force increment per voltage does not exceed 16.58%. Therefore, it can be concluded that the platform will be able to generate stable output force at different excitation voltage and excitation schemes while Videos S1 and S2, were included as a supplementary material in order to show platform operation with payload and without it.

## 5. Conclusions

A 2-DOF self-driving piezoelectric platform was developed and investigated. The structural design of the platform is simple and well scalable. Asymmetrical clamping of the bimorph plates allows achieve contact point motion in the YZ plane when vibrations of the first bending mode are excited. Such trajectory of motion induces normal and tangential forces to the contact surface and by this way generates planar motion of the platform.

Optimization of the length and width of the piezoelectric bimorph allowed us to increase vibration amplitudes of the contact point up to 508.12 µm while optimization study of the contact point location increased vibration amplitudes by 10.91%.

The design of the platform ensures low coupling between vibrations of the active and passive bimorph plates. Numerical investigation of the platform showed that the coupling ratio between passive and active piezoelectric bimorph plates is approximately 10 times. Therefore, coupling vibrations have a minor influence on the motion direction of the platform.

The experimental investigation confirmed the operation principle of the platform and showed that maximum planar motion velocity of 44.45 mm/s was achieved when excitation voltage of 210V_p-p_ and load of 55.68 g were applied. The maximum output force of the platform reached 44.16 mN at the same conditions.

## Figures and Tables

**Table 1:** Geometrical parameters of the platform.

Parameter	Value	Description
L_plate_	13 mm	Length of a rectangular plate
L_pzt_	8 mm	Length of a piezo ceramic plate
L_B_	7.03 mm	Distance between two thin junction beams
L_H_	7.65 mm	Distance between centers of two clamping holes
L_pos_	6.5 mm	Contact position along the bimorph
L	0.5 mm	Length of junction beam
W_Plate_	3 mm	Width of the rectangular plate
W_pzt_	3 mm	Width of piezo ceramic plate
W_pos_	2 mm	Contact position across the bimorph
W	0.25 mm	Width of junction beam
t_plate_	0.5 mm	Thickness of the rectangular plate
t_pzt_	0.2 mm	Thickness of piezo ceramic plate
t	0.9 mm	Thinness of the bimorph
D	1.65 mm	Diameter of clamping hole
S_D_	2 mm	Diameter of spherical contact
R	1.65 mm	Radius of triangle platform corners
α	60°	Angle between edges of the platform

**Table 2:** Switch positions for motion direction control.

Case No.	Motion	Direction	SW_1_	SW_2_	SW_3_
1	Forward	0°	1	0	0
	Backward	180°	0	1	1
2	Forward	120°	0	1	0
	Backward	300°	1	0	1
3	Forward	240°	0	0	1
	Backward	60°	1	1	0

**Table 3:** Properties of the materials used for numerical model.

Material Properties	Stainless Steel DIN 1.4301	PI Ceramics PIC155 [28]	Aluminium Oxide Ceramic
Density, [kg/m^3^]	8000	7750	3980
Young’s modulus, [N/m^2^]	10 × 10^9^	7.6 × 10^10^	41.9 × 10^10^
Poisson’s coefficient	0.3	-	0.33
Isotropic structural loss factor	0.02	-	0.2 × 10^−3^
Relative permittivity	-	ε_11_^T^/ ε_0_ = 1400 ε_33_^T^/ε_0_ = 1550	-
Elastic compliance coefficient [10^−12^ m^2^/N]	-	S_11_^E^ = 15.6 S_33_^E^ = 19.7	-
Elastic stiffness coefficient c_33_^D^, [N/m^2^]	-	11.1 × 10^10^	-
Piezoelectric constant d_33_ [10^−12^ m/V]	-	360	-
Piezoelectric constant d_31_ [10^−12^ m/V]	-	−165	-
Piezoelectric constant d_15_ [10^−12^ m/V]	-	540	-

**Table 4:** Summary of the results from optimization problems.

L_Plate_, mm	W_Plate_, mm	W_pos_, mm	u_tot_, µm
13	3	2	563.6

## Supplementary Materials

The following are available online at https://www.mdpi.com/article/10.3390/mi12111396/s1; Video S1: represents the operation of the platform under payload; Video S2: represents the operation of the platform without payload.
